# Impact of a group-based intervention program on physical activity and health-related outcomes in worksite settings

**DOI:** 10.1186/s12889-020-09036-2

**Published:** 2020-06-15

**Authors:** Ming Gu, Yejing Wang, Yan Shi, Jie Yu, Jiying Xu, Yingnan Jia, Minna Cheng

**Affiliations:** 1 School of Public Health, Key Lab of Public Health Safety of the Ministry of Education, Fudan University, 130 Dongan Road, Shanghai, 200032 China; 2 Huangpu District Center for Disease Control and Prevention, Shanghai, 200023 China; 3 Division of Chronic Non-communicable Disease and Injury, Shanghai Municipal Center for Disease Control and Prevention, 1380 West Zhongshan Road, Shanghai, 200336 China; 4 National Clinical Research Center for Aging and Medicine, Huashan Hospital, Fudan University, Shanghai, 200040 China

**Keywords:** Worksite, Physical activity, Intervention study, Group-based, Pedometer

## Abstract

**Background:**

The benefits of physical activity (PA) have been well documented, and the worksite is a promising setting for PA promotion. The aims of this study were as follows: 1. To evaluate the effect of a group-based worksite intervention on PA and health-related outcomes by using pedometers. 2. To examine the associations between the change in vigorous physical activity (VPA)/moderate physical activity (MPA)/walking and health related outcomes.

**Methods:**

A total of 398 participants (221 in the intervention group (IG) and 177 in the control group (CG)) from 17 worksites were recruited for a prospective self-controlled trial of a worksite physical activity intervention program in China. In the IG, a pedometer was utilized to self-monitor the PA, together with group competition, goal setting, and other incentives. No intervention was applied to the CG. Physical activity, sedentary behavior, and health-related outcomes were measured at baseline and immediately after the 100-day period intervention.

**Results:**

A total of 262 participants completed the program (68.3% adherence). Adherence in the intervention group was 67.9% (*n* = 150/221). Improvements between baseline and follow-up among intervention participants were observed in the following parameters: VPA (+ 109.7 METs/week; *p* < 0.05), walking (+ 209.2 METs/week; *p* < 0.01), systolic blood pressure (SBP; − 2.1 mmHg; *p* < 0.01), waist circumference (WC; − 2.3 cm; *p* < 0.01), body fat percentage (BF); − 1.0%; *p* < 0.01), and body mass index (BMI; − 0.5 kg/m^2^; *p* < 0.01). VPA was related to changes in body fat percentage (*p* < 0.05) and body mass index (*p* < 0.05).

**Conclusion:**

This integrated group-based intervention program contributed to comprehensive improvement in health-related outcomes. The study was useful for establishing associations between change in VPA/MPA/walking and health-related outcomes in a natural setting. Long-term evaluation is required to examine the potential of such an integrated intervention to promote PA.

**Registration:**

This study was prospectively registered in the Chinese Clinical Trial Registry. Trial registration number: ChiCTR-1,800,015,529. Date of registration: April 5, 2018.

## Background

The benefits of physical activity (PA) have been well documented. Regular PA reduces the risk of mortality [1], cardiovascular disease [1], type 2 diabetes [2], mental disorders [3], autoimmune diseases [4], and some types of cancer [5]. In addition, physical inactivity is the fourth leading cause of death worldwide [6]. More than 1.3 million deaths can be averted each year if the proportion of physical inactivity is decreased by 25% [7].

Despite the importance of PA, the occupational population had a lower level of PA compared to the retired crowd. The proportion of Chinese adults aged 20–59 years meeting the minimum recommendation of PA (150 min of moderate exercise per week or 75 min of vigorous exercise per week) was only 22.8% in 2014, while the proportion of obesity increased from 8.6% in 2000 to 12.9% in 2014 [8]. According to the China Kadoorie Biobank study, in which 466,605 Chinese adults were included, the mean level of exercise in those younger than 50 years (for women) and younger than 60 years (for men) was lower than that in those aged 70–79 years [9].

The worksite is a promising setting for PA promotion for the following reasons: the occupational population spends more than one-third of their waking hours at the worksite [10]; about 31% of workers reported that they had an opportunity to perform PA at the worksite [11]; compared with community residents, the occupational population is more stable and organized, which is easy for PA intervention and follow-up [12, 13], and PA programs at worksites have achieved success in improving health [14]. Therefore, PA interventions for the occupational population should focus on their workplaces.

A pedometer, a simple instrument to record the step data, is widely used for increasing the PA and motivating behavior change [15–21]. Evidence from a systematic review has shown that PA interventions at worksites can be effective [22]. Mixed findings regarding the effectiveness of pedometer-based interventions at the worksite were revealed in previous studies. Dadaczynski, K et al. [21] tested the effect of a gamified and pedometer-based intervention at the worksite on increasing the PA. In this randomized controlled trial of 144 participants, significant improvement was found in walking from 401 mins/week to 526 mins/week, but not in moderate-intensity physical activity or vigorous-intensity physical activity. Baghianimoghaddam et al. (2016) found similar results of a pedometer-based program in female employees in Iran [20]. Chae, D. et al. (2015) found that 39 of 70 participants completed the goal of “3,000 more steps” and experienced a significant decrease in BMI from 22.7 units to 22.4 units in a worksite pedometer-based intervention [19]. A systematic review found that four worksite pedometer-based PA interventions accompanied by individualized goal setting and weekly e-mail feedback caused small increases in PA and health-related outcomes [23]. The findings were similar to those in another review, which included four studies on pedometer-based intervention to increase the PA in a total sample of 1809 participants, and they revealed that there was a lack of sufficient evidence to evaluate the effectiveness of pedometer interventions for increasing the PA and improving health-related outcomes at the worksite. Freak-Poli suggested that a pedometer alone may not be effective enough in increasing a set of outcomes, and the type of intervention should be diversified in future studies [24].

Evidence has emphasized the significance of PA intensity on health-related outcomes. Clinical exercise trials have shown an association between vigorous-intensity physical activity (VPA) and cardiorespiratory fitness [25, 26]. Compared to VPA, moderate-intensity physical activity (MPA) has a greater effect on body composition [27]. This may be because it is easier to perform MPA continuously than VPA, and caloric expenditure and not PA intensity determines the changes in body composition [28]. However, all these results were found in clinical exercise trials, and in a natural setting, there is limited evidence regarding the effects of PA intensity on health-related outcomes [29]. Several studies on the pedometer-based PA intervention at the worksite have only focused on the primary outcomes like changes in PA or daily steps [20, 21, 30]. Only a few studies observed significant changes in PA and health-related outcomes, such as waist circumference (WC), body fat percentage (BF), and body mass index (BMI) [17, 19, 31, 32]. However, the relationship between PA intensity and health-related outcomes was not examined in these studies. Although it is better to perform any PA than not performing any PA [33], different effects of different PA intensities should be clarified so that PA can be more effectively promoted in the population.

A pedometer-based PA intervention was designed for an individual or a group according to previous studies. A worksite pedometer-based PA intervention study found that group participants experienced a larger increase in steps than individual participants [34]. Our intervention was designed for a group and it was based on the Social cognitive theory (SCT). This theory emphasizes that individual behavior is determined by the interaction among personal, environmental, and behavioral influences. The five constructs of the SCT are self-efficacy, socio-structural factors, outcomes expectations, goals, and behavior [35]. Socio-structural factors were emphasized in the group-based intervention: it reflected the typical Chinese culture and the characteristics of local occupational people; social norms were established and strengthened by intra-group supervision and support, which facilitated improvement of PA and health- related outcomes of individuals. A pedometer was applied to the intervention group as a way for improving the self-efficacy. To achieve a better effect, the intervention was designed for a group in which the participants could obtain social support. Combined with grouping and goal setting, individual and group incentives were provided to motivate the participants to perform active PA. The aims of this study are as follows: (1) to evaluate the effect of this group-based PA intervention by using pedometers at the worksite in China and (2) to examine the associations between changes in VPA/MPA/walking/sedentary time and health-related outcomes.

## Methods

### Study design and sample

A comprehensive PA intervention program was conducted in the Huangpu district of Shanghai, China from May 2018 to August 2018. Samples for the intervention group (IG) and control group (CG) were recruited from the same worksites. The study was performed in accordance with the Declaration of Helsinki. The research was approved by the ethics committee of the Fudan University School of Public Health China (IRB00002408&FWA00002399). At first, 17 worksites were screened for inclusion by connecting with the leaders at worksites. Participants in each worksite were recruited into the IG or the CG based on their willingness, and a written informed consent statement form was signed after recruitment. The inclusion criterion for participants was as follows: no willingness to resign within a year. The exclusion criterion for participants was as follows: presence of heart disease, cerebrovascular disease, mental illness, or physical disorders. The study was a 100-day intervention with measures taken at t1 (pre-intervention) and t2 (post-intervention). A total of 398 persons from 17 worksites participated in the study at baseline (IG = 221, CG = 177), and among them, 262 participants (68.3%) (IG = 150, CG = 112) completed the measures at baseline and final follow-up, with a 31.7% participant loss to follow-up. There was no significant difference in adherence between the two groups. Thus, the final sample comprised 262 persons (IG = 150, CG = 112) (Additional file 1). Chi-square tests showed no significant differences in the socio-demographic data between the final sample and participants lost to follow-up (Additional file 2).

The participants in the CG were only required to complete the measures. No intervention was applied to the CG.

### Intervention

The program included signing up for the groups. The number of participants in each group was 10–20, with a total of 47 groups. Each group chose one person as the group captain. Participants in the IG were required to wear a pedometer (Beijing Wanbu Health Technology Co., model: TW736) during all waking hours, except for hours in water (bathing and swimming) or in bed at night during the intervention period. Participants in the IG were instructed to upload the number of daily steps.

All participants in the IG were informed about the intervention at the beginning of the program, which included self-monitoring by a pedometer (Steps were measured and displayed on the pedometer. Participants in the IG were required to wear a pedometer on the waist during all waking hours, except for hours in water or in bed at night during the intervention period, and they were asked to upload the data to a specified website by connecting to a computer with a USB before September 16, 2018.), goal setting (10,000 steps/day and three walking prescriptions: the walking pace of participants was required to be 100–150 steps/minute; the first two prescriptions should last 10 min respectively; the third one should last 15 min), and competition among individuals and groups for achieving scores. More details about the scoring rules and walking prescriptions can be found in the Additional file 2. The personal total score was the sum of daily points. The sum of the personal score was divided by the number of group members, and the computed result was considered the score of each group. At the end of the program, the prizes and cash, as incentives, were evaluated by the individual and group scores for the IG.

#### Group incentives

First, a We-Chat group was created by each group captain to share the number of daily steps and to supervise and communicate with each other to achieve the corresponding goals. Group captains were required to motivate members to meet their daily goals and earn scores. In the We-Chat group, members would make an appointment to go running together after work, talk about personal scores, and so on. Second, according to the scoring rules, the top 3 scoring teams received prizes worth 2000 RMB at the end.

#### Individual incentives

Participants in the IG were encouraged to walk more than 10,000 steps and complete three walking prescriptions every day. According to the scoring rules, the top 10% scoring participants received prizes worth 200 RMB at the end of the 100-day intervention.

### Measures

Participants in the IG and CG were asked to complete the measures at baseline and the final follow-up. Demographic variables and health behavior data were collected in the self-reported questionnaire. The height variables were collected by a self-report, and the other health-related variables were measured by trained professionals.

### Demographic variables

Self-reported demographic variables included age, gender, marital status, and education. Four age categories were 18–29 years, 30–39 years, 40–49 years, and ≥ 50 years. The two marital statuses were married and unmarried/divorced/widowed. Four education categories were high school/technical, secondary school, junior college, and bachelor and master’s/doctorate degree.

### Health behavior

Three health behaviors and two potential factors were included in the study. Data on the current smoking status (current smoker, ex-smoker, or non-smoker), alcohol intake (weekly, monthly, or never) and PA were collected by using the self-report questionnaires. Participants were classified by their smoking status as non-smoker, quitting smoking during intervention, starting smoking or relapsing during intervention, and continuing smoking. For their drinking status, participants were divided into having no wine in the past year, quitting drinking during intervention, starting drinking during intervention, and continuing drinking. The Chinese version of the short International Physical Activity Questionnaire (IPAQ) was used to collect the data on PA, which was reliable (ICC of 0.79% and CV of 26%) [36]. The IPAQ includes three specific types of PA with respect to VPA, MPA, walking and sedentary time. The following three levels of PA were analyzed: VPA = 8.0 METs, MPA = 4.0 METs, and walking = 3.3 METs. Walking was considered as a type of moderate PA; however, the intensity of walking was slightly lower than that of moderate PA, such as jogging and dancing Total METs of each participant for these three types of PA and total sedentary time in the last 7 days were calculated.

Job demand and job control were based on the Karasek’s Job Content Questionnaire (JCQ) (Cronbach’s alpha was 0.88) [37]. Eleven Likert-scale items (scores ranging from 1 to 5) were used. A summary score of job demand and job control was constructed, wherein a high score indicated high job demand and job control. For questions related to job demand and job control, a score of 1 represented fully disagree, indicative of low job demand or job control; and a score of 5 represented fully agree, indicative of high job demand or job control. Job demand was measured by the following five items: working very fast, working very carefully, freedom from conflicting demands, using lots of information, and requests to complete excessive amounts of work. Job control was measured by the following six items: freedom to perform work, ability to make decisions, learning new things, a high level of skill, being creative, and doing non-repetitive work.

### Health-related outcomes

The measures of health-related outcomes included height, weight, Systolic Blood Pressure (SBP), Diastolic Blood Pressure (DBP), WC, Hip Circumference (HC), BF, and BMI. Height was calculated according to the self-reported data. All measures were performed by trained professionals using uniform methodology and instrumentation. Weight and BF were measured by a body fat meter (Beijing TONGFANG Health Technology Co., Model BCA-1C), which is based on the principles of bioelectrical impedance. Weight was measured allowing for a single layer of clothing, and it was recorded to the nearest 0.1 kg. Blood pressure was measured by an electronic sphygmomanometer (Citizen, Model PW332) after the participants remaining relaxed for two minutes. WC and HC were measured by a tape manually and recorded to the nearest 0.1 kg. WC was measured around the skin of the waist at the level of the umbilicus. HC was measured at the level of the pubic symphysis.

### Data analysis

Participants were classified into two groups; IG and CG. Descriptive analysis, chi-squared tests, and independent sample *t*-test were conducted to describe participant characteristics and the difference between IG and CG. Repeated measures analysis of variance with factors “group” and “time” was used to examine the impact of the intervention and time.

Multiple linear regression models were conducted to examine the factors that influenced changes in health-related outcomes. SBP, DBP, WC, Waist-to-Hip Ratio (WHR), BF, and BMI were analyzed using two models. Model 1 was used to evaluate the effects of intervention, including age, gender, baseline values of body composition, group, score difference of job demand, score difference of job control, smoking variables, and drinking variables. In Model 2, group variables were removed and the difference in METs for VPA/MPA/walking and the difference in sedentary time (mins/day) were introduced. This model was used to examine the associations between changes in VPA/MPA/walking and health-related outcomes. For all analyses, the level of significance was set at *p* < 0.05. Epidata 3.0.2, Excel 2016, and Statistical Package for Social Sciences 22.0 were used to conduct descriptive analyses, repeated measures ANOVA, and multiple liner regressions.

## Results

### Demographic characteristics (baseline)

Of the 398 participants, a total of 262 participants (IG = 150, CG = 112) completed both the baseline and final surveys, with a 34.2% loss to follow-up (IG = 32.1%, CG = 36.7%). Demographic characteristics are shown in Table 1. The final sample comprised 262 participants (IG = 150, CG = 112); Males (IG = 60.0%, CG = 50.0%) aged between 30 and 39 years (IG = 37.4%, CG = 38.4%) as well as a married status accounted for majority of the sample. Apart from the marital status, there were no significant differences between the IG and the CG in terms of the demographic characteristics.

**Table 1:** Demographic characteristics and health indicators of the participants at baseline

	Intervention group (IG) *n* = 150	Control group (CG) *n* = 112	*p*
	n(%)	n(%)	
Overall	150 (100)	112 (100)	
Gender			
Male	90 (60.0)	56 (50)	0.069
Female	60 (40.0)	56 (50)	
Age (years)			
18–29	29 (19.3)	25 (22.3)	0.634
30–39	56 (37.4)	43 (38.4)	
40–49	44 (29.3)	34 (30.4)	
≥ 50	21 (20.0)	10 (8.9)	
Marital status			
Married	115 (76.7)	69 (61.6)	**0.008**
Unmarried/Divorced/widowed	35 (23.3)	43 (38.4)	
Education			
High School/technical secondary school	22 (15.3)	20 (18.1)	0.071
Junior college	44 (30.6)	32 (29.1)	
Bachelor	55 (38.2)	52 (47.3)	
Masters/Doctorate	23 (16.0)	6 (5.5)	
Smoking status			0.393
Current smoker	25 (16.7)	24 (21.4)	
Ex-smoker	10 (6.7)	4.5 (5.0)	
Non-smoker	115 (76.7)	83 (74.1)	
Alcohol intake			0.106
Weekly	7 (4.7)	4 (3.6)	
Monthly	122 (81.3)	81 (72.3)	
Never	21 (14.0)	27 (24.1)	
	M, SD(±)	M, SD(±)	
Physical activity			
METs of vigorous-intensity PA	284.8 ± 702.9	362.5 ± 678.9	0.080
METs of moderate-intensity PA	159.5 ± 334.8	153.2 ± 294.7	0.875
METs of walking	955.5 ± 768.6	933.9 ± 794.5	0.825
Static time (mins)	308.1 ± 204.4	263.2 ± 205.7	0.080
Job demand	4.13 ± 0.65	4.00 ± 0.64	0.086
Job control	3.62 ± 0.59	3.5 ± 0.58	0.228
Systolic Blood Pressure (mmHg)	124.9 ± 20.1	122.5 ± 16.7	0.302
Diastolic Blood Pressure (mmHg)	78.4 ± 13.2	76.1 ± 11.2	0.131
Waist Circumference (cm)	85.1 ± 9.3	82.3 ± 9.8	**0.020**
Hip Circumference (cm)	97.7 ± 5.9	94.8 ± 6.2	**< 0.001**
Waist-to-Hip Ratio	0.88 ± 0.13	0.87 ± 0.06	0.247
Body Fat Percentage (%)	26.0 ± 5.0	25.4 ± 4.4	0.277
Body Mass Index (Kg/ m^2^)	23.7 ± 3.0	22.7 ± 3.1	**0.005**

### Health indicators (baseline)

At baseline, no significant difference was found in the smoking status, alcohol intake, METs for VPA/MPA/walking, sedentary time (mins/day), job demand, and job control between the IG and CG (Table 1). A significant difference was found in the WC, HC and BMI between the IG and CG. The average WC in the IG (85.1 ± 9.3 cm) was larger than that in the CG (82.3 ± 9.8 cm) at baseline. The IG (97.7 ± 5.9 cm) also showed greater values of HC than the CG (94.8 ± 6.2 cm) at baseline. The IG showed a larger BMI than CG at baseline.

### Changes in PA

Repeated measures ANOVA was used to evaluate the changes in PA. From t1 (284.8 mets) to t2 (394.5 mets), VPA in the IG changed significantly (*p* = 0.048), while no significant change was found in the CG (*p* = 0.23). It was surprising that METs for MPA decreased significantly in both the IG (*p* < 0.01) and the CG (*p* < 0.01). With respect to METs for walking, the IG showed an increase (*p* < 0.01) and no significant change was observed in the CG. There were no significant changes in the static time in the IG and CG (Table 2).

**Table 2:** Repeated-measures ANOVA and paired t-test for PA (M, SD(±))

PA		t1 M, SD(±)	t2 M, SD(±)	Time	Group x Time	Paired t-test
METs of VPA (METs)						
	IG	284.8 ± 702.9	394.5 ± 774.8	***p*** **= 0.03**	*p = 0.41*	***p*** **= 0.048**
	CG	362.5 ± 678.9	412.4 ± 775.8			*p* = 0.23
METs of MPA (METs)						
	IG	159.5 ± 334.8	66.4 ± 137.9	***p*** **< 0.01**	*P* = 0.80	***p*** **< 0.01**
	CG	153.2 ± 294.7	50.2 ± 91.2			***p*** **< 0.01**
METs of walking (METs)						
	IG	955.5 ± 768.6	1164.7 ± 894.3	***p*** **= 0.01**	***p*** **= 0.05**	***p*** **< 0.01**
	CG	933.9 ± 794.5	964.5 ± 821.2			*p* = 0.41
sedentary time (mins)						
	IG	308.1 ± 204.4	294.5 ± 212.4	*p* = 0.68	*p* = 0.27	*p* = 0.28
	CG	263.2 ± 205.7	269.4 ± 200.5			*p* = 0.61

### Changes in health-related outcomes

A repeated measures ANOVA (Table 3) was used to evaluate the effects of intervention. Significant decreases in SBP, WC, HC, BF, and BMI were found in the IG after intervention, while such decreases were not observed in the CG. The average SBP in the IG decreased from t1 (124.9 ± 20.1 mmHg) to t2 (122.3 ± 18.4 mmHg) (*p* < 0.01). For DBP, a significant increase was found in the CG from t1 to t2; however, it remained stable in the IG. There were significant decreases in WC (85.1 ± 9.3 cm at t1 to 82.3 ± 9.8 cm at t2) and HC (97.7 ± 5.9 cm at t1 to 94.8 ± 6.2 cm at t2) in the IG after the intervention period. The average BF was 26.0 ± 5.0% at t1. A significant decline in BF was 0.6% in the IG after the intervention period. In the CG, although a 0.2% increase in BF was observed, the change was not significant. There was a significant decrease in BMI in the IG from t1 (23.7 ± 3.0 Kg/m2) to t2 (22.7 ± 3.1 Kg/m2) (*p* < 0.01) (Table 3).

**Table 3:** Repeated-measures ANOVA and paired t-test for health-related outcomes (M, SD(±))

Outcomes		t1 M, SD(±)	t2 M, SD(±)	Time	Group x Time	Paired t-test
Systolic Blood Pressure (mmHg)						
	IG	124.9 ± 20.1	122.3 ± 18.4	*p* = 0.22	***p*** **< 0.01**	***p*** **< 0.01**
	CG	122.5 ± 16.7	123.6 ± 15.6			*p* = 0.18
Diastolic Blood Pressure (mmHg)						
	IG	78.4 ± 13.2	77.4 ± 11.8	*p* = 0.31	***p*** **< 0.01**	*p* = 0.15
	CG	76.1 ± 11.2	78.0 ± 10.8			***p*** **< 0.01**
Waist Circumference (cm)						
	IG	85.1 ± 9.3	82.8 ± 9.2	***p*** **< 0.01**	***p*** **< 0.01**	***p*** **< 0.01**
	CG	82.3 ± 9.8	82.6 ± 10.2			*p* = 0.33
Hip Circumference (cm)						
	IG	97.7 ± 5.9	95.0 ± 6.1	***p*** **< 0.01**	***p*** **< 0.01**	***p*** **< 0.01**
	CG	94.8 ± 6.2	95.4 ± 7.1			*p* = 0.07
Waist-to-Hip Ratio						
	IG	0.88 ± 0.13	0.87 ± 0.08	*p* = 0.2	*p* = 0.4	*p* = 0.18
	CG	0.87 ± 0.06	0.86 ± 0.08			*p* = 0.6
Body Fat Percentage (%)						
	IG	26.0 ± 5.0	25.0 ± 4.6	***p*** **< 0.01**	***p*** **< 0.01**	***p*** **< 0.01**
	CG	25.4 ± 4.4	25.6 ± 4.6			*p* = 0.09
Body Mass Index (Kg/m^2^)						
	IG	23.7 ± 3.0	23.2 ± 2.9	***p*** **< 0.01**	***p*** **< 0.01**	***p*** **< 0.01**
	CG	22.7 ± 3.1	22.8 ± 3.2			***p*** **= 0.047**

### Multiple linear regression analyses

Background variables (gender, age, education, and marital status) and independent variables (baseline values, score difference in job demand and control, smoking status and drinking status) were included in model 1(Table 4). Changes in the five health-related outcomes were related to the baseline values. Higher baseline values predicted a larger decrease in SBP (*p* < 0.001), DBP (*p* < 0.001), WHR (*p* < 0.001), BF (*p* < 0.001), and BMI (*p* = 0.005) after intervention. As expected, the IG showed a larger decrease in SBP (*p* = 0.005), DBP (*p* = 0.011), BF (*p* < 0.001), and BMI (*p* < 0.001) than the CG, but not in the WHR. With respect to change in SBP, those who quit smoking during intervention experienced a larger decrease than non-smokers (*p* = 0.026). Gender was significantly related to changes in WHR (*p* < 0.001), with females showing a larger decrease in WHR. There was a significant association between age and changes in BF (*p* = 0.025). As age increased, BF decreased more over time.

**Table 4:** Multiple Linear Regression Analyses of intervention effects for health-related outcomes: Model 1

	Systolic Blood Pressure Difference		Diastolic Blood Pressure Difference		Waist-to-Hip Ratio Difference		Body Fat Percentage Difference		Body Mass Index Difference	
	Std. Beta	*p*	Std. Beta	*p*	Std. Beta	*p*	Std. Beta	*p*	Std. Beta	*p*
		< 0.001		< 0.001		< 0.001		< 0.001		< 0.001
Age	−0.006	0.92	−0.020	0.74	0.076	0.09	−0.131	**0.025**	0.042	0.47
Males (Reference)										
Females	−0.105-	0.10	−0.114	0.08	−0.271	**< 0.001**	0.041	0.57	−0.026	0.67
Baseline Values	−0.435	**< 0.001**	− 0.446	**< 0.001**	− 0.772	**< 0.001**	−0.257	**< 0.001**	− 0.168	**0.005**
CG (Reference)										
IG	−0.165	**0.005**	−0.149	**0.011**	−0.020	0.65	−0.417	**< 0.001**	− 0.439	**< 0.001**
Score Difference of Job Demand	0.015	0.22	−0.035	0.60	−0.044	0.40	0.056	0.39	0.040	0.54
Score Difference of Job Control	0.031	0.65	0.007	0.92	0.050	0.34	−0.090	0.17	− 0.128	0.051
Non-smoker (Reference)										
Quitting Smoking during Intervention	− 0.050	0.39	−0.021	0.72	0.015	0.73	0.057	0.31	0.012	0.84
Starting Smoking or Relapsing during Intervention	0.009	0.16	−0.029	0.61	−0.002	0.97	0.044	0.43	−0.033	0.56
Continuing Smoking	−0.008	0.91	0.019	0.77	0.046	0.35	0.059	0.35	0.066	0.29
Having No Wine in the Past Year										
Quitting Drinking during Intervention	0.132	**0.026**	−0.014	0.81	−0.034	0.45	−0.005	0.92	0.091	0.11
Starting Drinking during Intervention	0.033	0.57	0.005	0.93	0.007	0.87	0.058	0.31	−0.072	0.21
Continuing Drinking	0.014	0.82	0.006	0.92	0.009	0.85	−0.015	0.80	0.084	0.15

Background variables (gender, age, education, and marital status) and independent variables (baseline values, METs difference in VPA, MPA and walking, difference in sedentary time, score difference in job demand and control, smoking status and drinking status) were included in model 2 (Table 5). Group variables were removed and the difference in METs for VPA/MPA/walking and the difference in sedentary time (mins/day) were introduced. This model was used to examine the associations between changes in VPA/MPA/walking and health-related outcomes. Like the results from model 1, higher baseline values predicted a larger decrease in SBP (*p* < 0.001), DBP (*p* < 0.001), WHR (*p* < 0.001), BF (*p* < 0.001), and BMI (*p* = 0.001) after intervention. There were significant gender differences in changes in WHR (*p* < 0.001). Females showed a larger decrease in WHR than males. Age (*p* < 0.027) and difference in METs for VPA (*p* < 0.001) were significantly related to changes in BF, with older participants and higher difference predicting a larger decrease in BF. Those who had a higher difference in METs for VPA showed a larger decrease in BMI (*p* = 0.013) (Table 5).

**Table 5:** Multiple Linear Regression Analyses of associations between changes in VPA/MPA/walking/sedentary time and health-related outcomes: Model 2

	Systolic Blood Pressure Difference		Diastolic Blood Pressure Difference		Waist-to-Hip Ratio Difference		Body Fat Percentage Difference		Body Mass Index Difference	
	Std. Beta	*p*	Std. Beta	*p*	Std. Beta	*p*	Std. Beta	*p*	Std. Beta	*p*
		< 0.001		< 0.001		< 0.001		< 0.001		0.057
Age	−0.012	0.84	− 0.033	0.59	0.069	0.13	−0.142	**0.027**	0.025	0.69
Males (Reference)										
Females	− 0.078	0.24	−0.095	0.14	−0.271	**< 0.001**	0.109	0.16	0.001	0.99
Baseline Values	−0.440	**< 0.001**	−0.458	**< 0.001**	− 0.765	**< 0.001**	− 0.318	**< 0.001**	−0.227	**0.001**
METs difference of VPA	0.031	0.61	0.111	0.06	−0.033	0.47	−0.167	**0.008**	−0.159	**0.013**
METs difference of MPA	0.012	0.84	−0.028	0.63	−0.053	0.23	−0.032	0.60	−0.039	0.53
METs difference of Walking	0.021	0.73	−0.093	0.12	−0.034	0.45	−0.029	0.64	−0.009	0.89
Difference of sedentary time (mins/day)	−0.047	0.43	−0.057	0.33	0.042	0.35	0.096	0.12	0.068	0.27
Score Difference of Job Demand	0.001	0.99	−0.078	0.26	−0.057	0.28	0.020	0.79	0.001	0.99
Score Difference of Job Control	0.035	0.62	0.017	0.80	0.058	0.27	−0.069	0.34	− 0.102	0.17
Non-smoker (Reference)										
Quitting Smoking during Intervention	−0.054	0.38	−0.024	0.69	−0.002	0.97	−0.006	0.93	−0.057	0.38
Starting Smoking or Relapsing during Intervention	0.001	0.99	−0.047	0.42	−0.007	0.87	0.002	0.97	−0.074	0.23
Continuing Smoking	0.018	0.78	0.045	0.49	0.047	0.33	0.106	0.12	0.120	0.08
Having No Wine in the Past Year (Reference)										
Quitting Drinking during Intervention	0.118	0.06	−0.061	0.32	−0.030	0.52	0.022	0.74	0.116	0.08
Starting Drinking during Intervention	0.008	0.90	−0.025	0.67	0.010	0.82	0.027	0.66	−0.106	0.10
Continued Drinking	0.019	0.76	0.017	0.78	0.001	0.99	−0.034	0.60	0.066	0.31

## Discussion

This group-based intervention was designed to improve PA and health-related outcomes in a sample of occupational population in Shanghai. Overall, comprehensive improvement in PA and health-related outcomes was observed. Compared to the CG, we found that a 100-day group-based PA intervention in the occupational population can help improve VPA, walking, SBP, BMI, WC, HC, BF, and BMI. Changes in BF and BMI were related to VPA.

The 100-day group-based intervention led to an increase in walking of about 22% compared to baseline. This result corresponded to the findings of a systematic review, in which an average 26.9% increase in walking was noted [23]. Compared to two recent studies, in which PA was evaluated by the IPAQ, our result was smaller than the increase of 30% in the study by Dadaczynski et al. [21] and 70% in the study by Baghianimoghaddam et al. [20] A significant increase in VPA was observed in our study; however, this result was not found in recent studies [20, 21]. There are three possible explanations for this occurrence. First, walking pace was highlighted in our study as a setting goal, while it was not mentioned in the other two studies. Second, due to the shortage of time, occupational participants might run to complete 10,000 steps/day in a short time, which is a type of VPA. Third, although the differences were not significant, the IG showed a lower level of VPA and more sedentary time than the CG at baseline. Also, it was noted in a systemic review that a pedometer-based intervention would be more effective in improving PA when participants had a PA-inactive lifestyle [23]. Thus, the differences may have contributed to the increase in VPA. To our surprise, a significant decrease in MPA was found in both the IG and CG. This may be because the participants tend to be physically inactive during hot summer, which was the intervention period. And for completing the daily goal in a short time, participants in the IG chose VPA like running but not MPA.

In addition to the effects on PA, significant improvements in most health-related outcomes were revealed in the evaluation. A significant decrease was found in SBP to the order of − 2.6 mmHg in the IG. This improvement was consistent with findings from a review on worksite pedometer-based intervention studies reporting that the mean decrease in SBP was 3.11 mmHg [14]. A 2.3 cm significant decrease in WC was observed in the IG in our study. This change had its clinical implications for preventing central obesity [38]. Compared to four worksite pedometer-based PA individual intervention studies, which reported a decrease in WC ranging from 0.43 cm to 4.4 cm [17, 39–41], this was a moderate change. It was interesting to observe that participants in these studies had a higher average WC at baseline than those in our study, and a larger decrease in WC could be easily observed in participants of their studies. This may be one of the reasons why the decrease in WC in our study was not outstanding. In our study, a significant decrease of 0.5 units in BMI was observed. No significant improvements in BMI were observed in the three recent studies [17, 31, 39]. According to the physiology of weight loss, a significant decrease in BMI would be more likely when PA is performed at a high intensity and not at low-to-moderate intensities [23]. This finding was supported by the result that an increase in VPA was observed in our study. While no significant difference in BF between the IG and CG was found at baseline, the IG experienced a significant decrease in BF from 26.0 to 25.0% over the 100-day intervention period. The result was consistent with the finding in the study by Mansi, S., et al. [30], in which a 1.7% decrease in BF was reported after a 3-month walking program at the worksite. It suggested that walking pace contributed to this improvement [42]. The intervention approach of recent worksite pedometer-based PA intervention studies included goal setting (10,000 steps/day and other health goals), incentives (cash incentives for individuals), and feedback (personalized weekly emails) [21, 30, 31, 39]. Different from these studies, the comprehensive PA intervention program emphasized on grouping, walking pace goal (pace at 100–150 steps/minute), intra-group supervision and support, inter-group competition, and group incentives. In general, comprehensive improvements in most health-related outcomes were observed in our study.

In short, there were comprehensive improvements in health-related outcomes in the IG over the 100-day intervention period. This significant improvement was likely related to the design of the group-based intervention. In our intervention, the role of the group was highlighted by intra-group supervision and support, inter-group competition, and group incentives. Compared with individual-based intervention, group-based intervention has the following advantages: a group provides the following three categories of support: emotional, appraisal, and informational support, which encourage the group members to be persistent and optimistic about the health promotion programs [43]; and group spirit and interaction have the potential to lower disinterest and increase motivation [44, 45]. Further, because of the Chinese culture, Chinese people value collective consciousness and have a high concurrence of positive and negative emotions [46]. Thus, it was suggested that a group-based intervention would be a good way to improve PA at the worksite in China. These elements, together with a pedometer, goal setting, and individual incentives, were integrated into our worksite health promotion program. While a pedometer alone may not be effective to improve the health-related outcomes, this intervention mode showed a comprehensive effect in Chinese occupational persons. Meanwhile, a systematic review suggested that health was related to walking pace rather than walking time and change in body composition was mainly related to the increase in MVPA [47]. This finding was also supported by the results of our study, and it was suggested that walking pace should be considered in a pedometer-based PA intervention at the worksite.

From analyses of associations between changes in VPA/MPA/walking and health-related outcomes, it was found that higher BMI values at baseline and improvement in VPA predicted a greater decrease in BMI in the IG. Like the change in BMI, higher BF values at baseline and improvement in VPA were indicators of a larger decrease in BF during the intervention. The finding was consistent with that in previous studies, which suggested that low-to-moderate intensity PA did not contribute to the reduction in body composition due to the physiology of weight loss but VPA contributed to the reduction in body composition [16, 23]. However, another study suggested that MPA had a greater effect on body composition compared VPA [27]. This finding should be examined in future study, while investigating the association between PA intensity and health-related outcomes in a natural setting like a worksite. A study found that most people with higher BMI or BF are physically inactive [48]. Our study also suggested that the PA intervention should be focused on those with high BMI or BF, who would have greater improvement in body composition.

This was the first group-based worksite PA intervention with a pedometer in China, which was the main strength of our study. An integrated intervention mode contributed to comprehensive improvement in health-related outcomes compared to that in most previous studies. The study was useful in establishing associations between PA intensity and health-related outcomes in a natural setting. This study provided evidence for promotion of PA intervention at a worksite in China.

However, there are some limitations to this study. First, the rates of loss to follow-up in the IG and CG were high. One of the reasons for this occurrence was scattered locations of the survey. The other reason was the conflict between investigation time and working time, which forced the participants to refuse the investigation at follow-up. Second, there was lack of information on dietary habits, which were important factors for some health-related outcomes, such as WC, BMI, and BF. Third, this study evaluated the short-term effects of the intervention at worksites, and it did not evaluate the lifestyle behavioral changes in individuals and long-term maintenance. Fourth, we used the IPAQ to evaluate PA, and therefore, there was a lack of objective data.

## Conclusion

This integrated group-based intervention program contributed to comprehensive improvement in health-related outcomes. The study was useful for establishing associations between the change in VPA/MPA/walking and health-related outcomes in a natural setting. Long-term evaluation is required to examine the potential of such an integrated intervention to promote PA.

## Supplementary information

**Additional file 1.** CONSORT diagram of participants flow through this program.

**Additional file 2 Table S1.** Comparison of demographic characteristics of follow-up and lost. **Table S2.** Scoring rules for individuals.

## Data Availability

The data that support the findings of this study are available from the corresponding author, Mr. Yingnan Jia.

## Abbreviations

PA: Physical activity
VPA: Vigorous physical activity
MPA: Moderate physical activity
MVPA: Moderate-to-vigorous physical activity
IG: Intervention group
CG: Control group
IPAQ: International physical activity questionnaire
SBP: Systolic blood pressure
DBP: Diastolic blood pressure
WC: Waist circumference
HC: Hip circumference
BF: Body fat percentage
BMI: Body mass index
SCT: Social cognitive theory
