# Supplementary figures and images for: Impact of a group-based intervention program on physical activity and health-related outcomes in worksite settings
Source: BMC Public Health. 2020 Jun 15;20:935. doi: 10.1186/s12889-020-09036-2 (PMC7294670; doi:10.1186/s12889-020-09036-2)

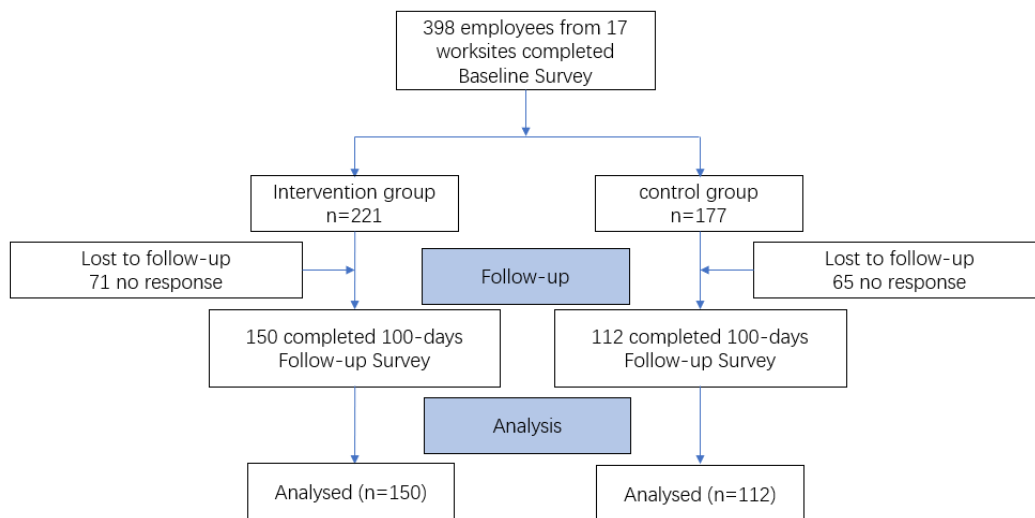

CONSORT diagram of participants flow through this program

Supplement: Supplementary file 1 — Additional file 1. CONSORT diagram of participants flow through this program. [file 12889_2020_9036_MOESM1_ESM.pdf]
