# Supplementary material for: Impact of a group-based intervention program on physical activity and health-related outcomes in worksite settings
Source: BMC Public Health. 2020 Jun 15;20:935. doi: 10.1186/s12889-020-09036-2 (PMC7294670; doi:10.1186/s12889-020-09036-2)
Supplement: Supplementary file 2 — Additional file 2 Table S1. Comparison of demographic characteristics of follow-up and lost. Table S2. Scoring rules for individuals. [file 12889_2020_9036_MOESM2_ESM.pdf]

Table S1. Comparison of demographic characteristics of follow-up and lost

	follow-up<br>n=260<br>n (%)	lost<br>n=136<br>n (%)	*p*
Gender			
Male	146(55.7)	80(58.8)	>0.05
Female	116(44.3)	56(41.2)	
Age (years)			
18-29	54(20.6)	39(28.7)	>0.05
30-39	99(37.8)	46(33.8)	
40-49	78(29.8)	36(26.5)	
≥50	31(11.8)	15(11.0)	
Marital status			
Married	184(70.2)	89(65.4)	>0.05
Unmarried/Divorced /widowed	78(29.8)	47(34.6)	
Education			
High School/technical secondary school	42(16.0)	18(13.2)	>0.05
Junior college	84(32.1)	32(23.5)	
Bachelor	107(40.8)	61(44.9)	
Masters/Doctorate	29(11.1)	25(18.4)	
Overall	262(100)	136(100)	

Table S2. Scoring rules for individuals

Items	Score
①**Daily steps**	
6,000-7,999	1
8,000-9,999	2
≥10,000	3
②**Walking prescription** (pace at 100-150 steps/minute)	
completing the first prescription (lasts 10 minutes)	1
completing the first two prescriptions (each lasts 10 minutes)	3
completing three prescriptions (the third lasts 15 minutes)	6
③**Completing 3,000 steps in 05:00-09:00 or 4,000 steps in 17:00-23:00**	1
